# Preliminary validation of the Brazilian version of SPRouT for screening reading difficulties

**DOI:** 10.1590/2317-1782/e20250165en

**Published:** 2026-07-08

**Authors:** Beatriz de Souza dos Reis França, Lucas Alves Maia, Ricardo Franco de Lima, Cíntia Alves Salgado-Azoni

**Affiliations:** 1 Programa Associado de Pós-graduação em Fonoaudiologia UFRN/UNCISAL/UFPB – PPgFon, Universidade Federal do Rio Grande do Norte – UFRN - Natal (RN), Brasil.; 2 Universidade Federal do Rio Grande do Norte – UFRN - Natal (RN), Brasil.; 3 Programa de Pós-graduação em Psicologia, Universidade São Francisco – USF - Campinas (SP), Brasil.

**Keywords:** Screening, Reading, Dyslexia, Literacy, Psychometrics

## Abstract

**Purpose:**

To investigate preliminary evidence of validity based on relations to external variables for the Brazilian version of the Screening Pediatric Patients for Reading Difficulties Test (SPRouT).

**Methods:**

Following approval by the Research Ethics Committee, 27 children aged four to eight years were individually assessed using the SPRouT and standardized instruments measuring intelligence and phonological processing skills. Analyses included Spearman correlations (controlling for intelligence) and simple linear regressions to examine the predictive capacity of the SPRouT.

**Results:**

Moderate to strong negative correlations were observed between SPRouT scores and measures of phonological awareness, phonological working memory, and rapid naming. The total SPRouT score explained between 23% and 32% of the variance in phonological measures, with medium to large effect sizes.

**Conclusion:**

Findings provide initial evidence of convergent validity for the SPRouT, as it demonstrated associations with skills related to reading development. Regression analyses also suggest its predictive potential, although longitudinal studies and clinical samples are needed to confirm this property. The SPRouT represents a promising tool for screening reading difficulties, and further psychometric investigations with larger and more diverse samples are recommended.

## INTRODUCTION

The acquisition of reading is an essential milestone in child development, fundamental for academic success and full social participation. Before the start of formal education, the development of precursor reading skills is indispensable, as they establish the foundations for subsequent reading proficiency^(1,2)^.

Several studies have investigated the components of phonological processing, highlighting their contributions to the acquisition and development of reading^(3,4)^. Among these components, phonological awareness stands out as a strong predictor of reading performance, considered an important competence to be included in screening instruments for reading difficulties. This skill allows children to distinguish and manipulate speech sounds, consequently facilitating the reading process^(5)^.

In turn, rapid automatic naming, often measured by the speed of naming familiar visual stimuli, has proven to be a stable marker throughout development, with strong predictive capacity for reading^(6)^. Furthermore, phonological memory, usually assessed by tasks with digits or pseudowords, has also been significantly related to initial reading, acting as a predictor of early success in learning to read^(7)^.

A systematic review conducted by Schoenel et al.^(8)^ found that children with difficulties in the literacy process perform worse on several phonological processing skills than those with adequate literacy. This data reinforces the importance of phonological processing as a key predictor for the development of reading and writing.

However, the challenges associated with reading difficulties and their early assessment remain significant, despite knowledge of these skills and the impact of reading on development. Late identification of reading difficulties can have significant consequences on children’s academic performance and socio-emotional development, such as demotivation, low self-esteem, and learning difficulties, which can persist throughout adulthood^(9)^.

In Brazil, the results of the Basic Education Assessment System (Saeb 2021) show persistent challenges in Portuguese proficiency among 2nd graders. More than half of the students assessed were classified at the lowest levels of the proficiency scale that evaluates mastery of the alphabetic principle, reading skills, and text production^(10)^. This scenario highlights the need to improve the identification of children at risk for literacy development, as well as effective interventions.

In the clinical setting, pediatricians can play an important role in screening for reading difficulties through analysis of family history and use of validated and standardized screening tools^(11)^. Although early identification faces challenges regarding accuracy, its application allows for early interventions, which can prevent or mitigate reading difficulties before they become established^(12)^.

For screening instruments to be used with scientific rigor and clinical applicability, their psychometric properties must be systematically investigated. As established by the Standards for Educational and Psychological Testing^(13)^, the validity of an instrument must be supported by multiple sources of evidence, namely: (1) evidence based on content, (2) on internal structure, (3) on the relationship with external variables, (4) on response processes, and (5) on the consequences of using the test.

Among these, evidence based on relationships with external variables refers to the degree to which test scores are associated with other measures or outcomes, in a manner consistent with the construct that is intended to be evaluated^(13)^. This type of evidence is particularly relevant in initial validation studies, as it verifies the theoretical coherence and practical usefulness of the instrument when correlated with established measures.

Despite the importance of this type of evidence, validation studies for screening and assessment measures of child development are scarce in the Brazilian context^(14)^. This limitation indicates not only the need for greater rigor in the selection and use of these instruments but also reinforces the importance of fostering research aimed at validating them to provide more comprehensive and reliable tools for identifying developmental risks.

Thus, the Screening Pediatric Patients for Reading Difficulties Test (SPRouT) was originally developed from a clinical perspective to meet the practical needs and dynamic routine of pediatricians. It is a brief checklist, not yet validated, to assess reading difficulties in children aged 4 to 8 years^(15)^. Its translation and cross-cultural adaptation have already been explored for both Brazilian Portuguese (named Brazilian Version of the Screening Pediatric Patients for Reading Difficulties Test)^(16)^ and Spanish (named *Prueba pediátrica de detección de difícildes de alfabetización: Borrador* [Pediatric test for detecting literacy difficulties: Eraser] - PEDAL-B)^(17)^. However, studies investigating its psychometric properties are still needed.

Considering such aspects, this study aimed to investigate preliminary evidence of the validity of the SPRouT based on its relationship with other variables, specifically through the association between its scores and measures of phonological skills known to be associated with reading development.

## METHODS

### Study type and ethical considerations

This descriptive, analytical, cross-sectional, methodological study consists of a preliminary validation stage of the screening instrument, the Brazilian Version of the SPRouT, based on the stages of construction, translation, and cross-cultural adaptation already completed^(16)^.

This study was approved by the Research Ethics Committee (CEP) of the University of São Francisco (USF), under opinion number 6.042.270, in accordance with the resolution of the Brazilian National Health Council - CNS 466/12 on Guidelines and Regulatory Standards for Human Research. All participants signed an informed consent form.

### Participants

This study included children aged 4 to 8 years, Portuguese speakers, enrolled in private schools, with or without complaints related to oral and/or written language, as reported by parents or legal guardians. The inclusion of children with complaints was justified by the higher risk of presenting learning disorders. The exclusion criterion was the withdrawal of participants during the evaluation process.

The study included 27 children and their parents/guardians, aged 52 to 106 months (Mean = 76.89; SD = 16.05), of both sexes, being 13 boys (48%) and 14 girls (52%). They attended preschool (n = 7; 26%), kindergarten (n = 5; 19%), 1^st^ grade (n = 9; 33%), 2^nd^ grade (n = 3; 11%), and 3^rd^ grade (n = 3; 11%). All children attended a private school in a city in Northeast Brazil.

In the total group, 19 children (70%) did not have diagnoses, while eight children (30%) had specific suspected diagnoses, as reported by their parents. Among these, conditions such as autism spectrum disorder (ASD), childhood apraxia of speech (CAS), phonological disorder, and attention-deficit/hyperactivity disorder (ADHD) stood out.

Although SPRouT was developed for clinical use in pediatric patients, its application in an educational setting was prioritized due to the availability of the sample and researchers. This methodological choice may contribute to its use in different contexts, but it represents a limitation regarding its suitability for the original target audience.

### Materials

From a psychometric standpoint, to meet the study's objectives, instruments widely used in clinical and educational contexts were selected, all standardized for the Brazilian population and with evidence of validity. These instruments assess different components of phonological processing, in addition to intelligence.

#### Brazilian version of the Screening Pediatric Patients for Reading Difficulties Test (SPRouT)^(16)^

The instrument consists of five forms (A-E), selected based on the child's age. Each form has two sections: one with questions regarding the child's clinical and developmental history to be completed with the parents, and another with items intended for a quick clinical assessment with the child. The clinical section may contain five to eight items, varying according to the form used. The tasks in this section assess linguistic skills considered prerequisites for reading, such as phonological awareness and phonological working memory^(16)^. For assessment purposes, scores are assigned to items that indicate errors or signs of possible difficulties in development, speech, and learning. This study used the total raw score, which is the sum of these indicators. Thus, the higher the score, the greater the risk of the child having reading difficulties.

#### Phonological Awareness Test through Oral Production - PCFO^(18)^

This test assesses phonological awareness skills through 10 subtests: syllabic and phonemic synthesis, judgment of rhymes and alliterations, segmentation, manipulation, and transposition of syllables and phonemes. Each subtest has, in addition to the instruction, two training items and four test items. One point is awarded for each correct answer. The PCFO should be administered individually, and the average administration time is 20 minutes. The raw score of all subtests, which corresponds to the total number of correct answers, is converted into a standard score for classification purposes. Reliability studies have identified indices ranging from 0.86 to 0.91 for the PCFO, depending on the method used (internal consistency and split-half method), in samples of preschool to elementary students^(18)^.

#### Word and Pseudoword Repetition Test - TRPP^(19)^

This test assesses phonological short-term memory through a word and pseudoword repetition task. The examiner pronounces a sequence of two to six words to the child, with a 1-second interval between them, and the child's task is to repeat the words in the same sequence. There are two sequences for each length (words and pseudowords). The test is administered individually, with 1 point awarded for each correct sequence, which is obtained in a standard score for classification. The average administration time is 7 minutes. Regarding reliability, studies have identified indices ranging from 0.77 to 0.83 for TRPP, considering the internal consistency and split-half methods. In addition, the test-retest method indicated a correlation index of 0.73^(19)^.

#### Automatized Naming Test - TENA^(20)^

The test individually assesses the ability of rapid automatic naming, consisting of four subtests: naming of colors, objects, letters, and numbers. Each subtest contains five stimuli, which are randomly repeated 10 times in each of the five lines, totaling 50 stimuli per board. The examiner asks the subject to name each stimulus as quickly as possible, without mistakes, both on the training board and on the test board. During the assessment, the number of errors and the total naming time in each subtest are recorded^(20)^. As this is a standardized instrument for the Brazilian population, the percentiles of time and errors for each subtest were considered. Reliability studies, based on temporal stability, demonstrated significant positive correlations with magnitudes ranging from moderate to high between applications, especially in time scores.

#### Columbia Mental Maturity Scale - CMMS^(21)^

This instrument is designed to assess fluid intelligence (Gf), does not require oral responses, is for the exclusive use of psychology professionals, and has been approved by the Psychological Tests Assessment System (SATEPSI, in Portuguese). It consists of 92 pictorial and figurative classification items, organized into eight levels according to the child's age range. This study used the total score of correct answers, expressed as a percentile. Brazilian studies report CMMS accuracy rates ranging from 0.86 to 0.93.

### Procedures

#### Recruitment

The participant recruitment process was carried out with the assistance of the school's administrative office, which collaborated in identifying active students from the institutional database. The parents/guardians of students within the age range compatible with the study's inclusion criteria were initially contacted via phone calls.

**Figure 1:**
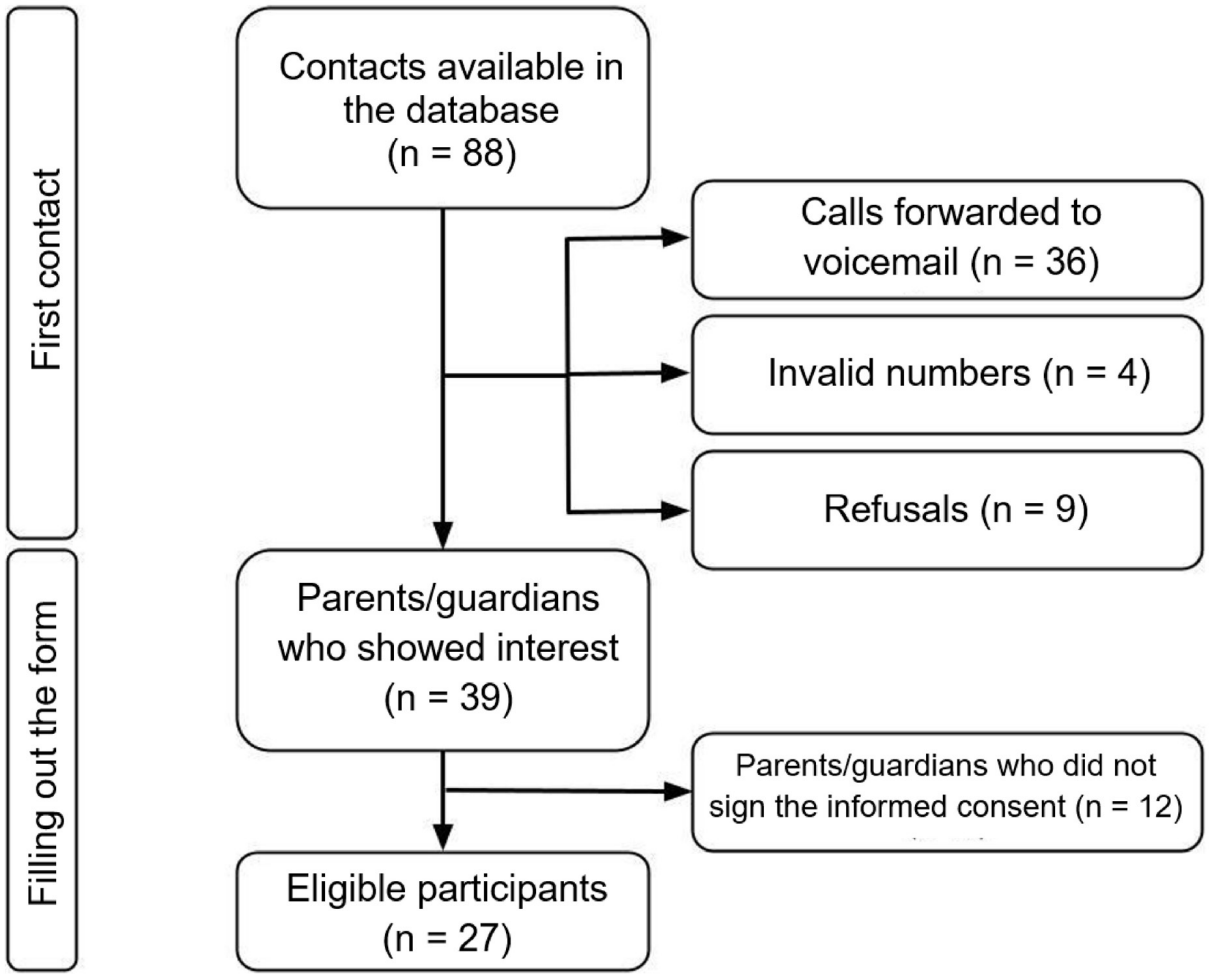
Flowchart of the participant recruitment process

Among the main reasons for their refusal, the occurrence of ongoing therapeutic follow-up with the child stood out. Parents/guardians who expressed interest in participating in the study were digitally directed to a form containing detailed information about the child's clinical history and an informed consent form. In addition, supplementary informational material, developed by the authors using the Canva platform, was sent in PDF via the WhatsApp Business messaging application. Figure 1 presents a flowchart of the recruitment process, from initial contact to the selection of the final sample.

#### Assessment

Data were collected by convenience sampling in two units of a private school in a city in northeastern Brazil, selected due to the feasibility of access for the researchers. The process took place in July and August 2024. The first unit consisted of preschool, kindergarten, and first-grade students, while the second was intended for second grade up to high school.

The assessments took place in the school units during class time, previously agreed upon with the pedagogical team, so as not to interfere with the students' academic performance. An interdisciplinary team (four speech-language-hearing pathologists, an undergraduate speech-language-hearing student, a psychologist, and an undergraduate psychology student), calibrated before beginning data collection, applied the instruments in rooms with adequate acoustic and visual conditions to ensure the concentration and comfort of the participants.

Due to physical space limitations, the groups were organized with a maximum of four children per room, each accompanied by a researcher. The assessments lasted an average of 60 minutes per child. In some cases, it was necessary to divide the time into two sessions, due to the requirement that the CMMS be administered by a psychology professional. Furthermore, not all children were able to complete all the instruments, due to attentional difficulties or lack of cooperation during data collection.

#### Data analysis

Descriptive analyses were performed to characterize the sample with respect to sociodemographic variables, including age, sex, and education level. Descriptive statistics (mean, standard deviation, minimum, and maximum values) were calculated for continuous variables, while absolute and percentage frequencies were reported for categorical variables. The number of participants in the statistical analysis varied due to incomplete or invalid responses. These variations resulted from difficulties in understanding or cooperating on the part of some children during data collection.

Considering that the test scores did not have a normal distribution (Shapiro-Wilk; p < 0.05), Spearman's correlation analysis was conducted to investigate the relationships between the total SPRouT scores and the instruments that assess phonological processing. As variations in intelligence level can influence performance on phonological tasks, it was decided to control for this factor in the analyses. Thus, the CMMS percentile was used as a control variable to isolate the effect of general intelligence and verify more precisely the relationship between the SPRouT and the phonological skills assessed. The correlations were interpreted according to Cohen’s magnitude criteria^(22)^, classified as weak: between 0.10 and 0.29; moderate: between 0.30 and 0.49; and strong: between 0.50 and 1.

A linear regression analysis was performed to assess the predictive potential of the total SPRouT score in relation to the percentiles of the tests that assess phonological processing. Thus, the total SPRouT score was considered as the independent variable, while the percentiles of the instruments (PCFO, TRPP, and TENA) were defined as dependent variables. The criteria for evaluating the model included coefficient of determination (R^2^): to measure the proportion of variance explained by the independent variable; Statistical significance (p): p < 0.05 was considered significant; Effect size (f^2^): calculated using the formula f^2^ = R^2^/(1−R^2^), interpreted as: small: f^2^ ≥ 0.02, medium: f^2^ ≥ 0.15, and large: f^2^ ≥ 0.35.

## RESULTS

Table 1 presents the descriptive analysis of the instruments. The CMMS classifications were distributed as follows: extremely low (n = 1; 4%), very low (n = 1; 4%), average (n = 19; 70%), and above average (n = 6; 22%).

**Table 1:** Descriptive statistics of the scores from the administered instruments

**Scores**		**N**		**M**		**SD**		**Min.**		**Max.**	
SPRouT (history)		26		3.73		1.95		1.00		8.00	
SPRouT (clinical)		26		6.15		4.49		0.00		16.00	
SPRouT (total)		26		10.00		5.58		2.00		24.00	
TRPP (words)		26		104.92		21.12		75.00		145.00	
TRPP (pseudowords)		24		106.63		23.19		82.00		149.00	
TRPP (total)		26		104.89		22.16		69.00		155.00	
PCFO (total)		27		110.41		37.78		61.00		211.00	
TENA Color (time)		26		44.81		26.63		10.00		90.00	
TENA Color (error)		22		39.32		27.53		10.00		90.00	
TENA Obj (time)		26		46.54		29.39		10.00		90.00	
TENA Obj (error)		20		33.50		25.86		10.00		90.00	
TENA Letter (time)		22		44.77		29.86		10.00		90.00	
TENA Letter (error)		19		35.53		27.43		10.00		90.00	
TENA Num (time)		22		42.27		31.84		10.00		90.00	
TENA Num (error)		19		28.68		25.92		10.00		90.00	
CMMS		27		57.96		21.97		1.00		90.00	

**Caption:** N = sample; M = Mean; SD = standard deviation; Min. = Minimum; Max. = Maximum

According to Table 2, the results indicated significant negative correlations between the SPRouT and most instruments (TRPP pseudoword subtest, TRPP total score, PCFO, TENA color - time, and TENA letter - time and error). The correlations ranged from weak (rho = -0.174) to strong (rho = -0.804).

**Table 2:** Spearman correlation between SPRouT and the other instruments

	**Spearman's rho**	***Magnitude***	***p***	**Lower 95% CI**	**Upper 95% CI**
TRPP_Rep_words	-0.326	Moderate	0.120	-0.674	0.089
TRPP_Rep_pseudo	-0.484	Moderate	0.022*	-0.777	-0.096
TRPP	-0.524	Strong	0.009*	-0.796	-0.183
PCFO	-0.719	Strong	< .001*	-0.889	-0.408
TENA_Color_Time	-0.494	Moderate	0.014*	-0.770	-0.119
TENA_Color_Error	-0.330	Moderate	0.155	-0.674	0.111
TENA_Obj_Time	-0.174	Weak	0.417	-0.533	0.211
TENA_Obj_Error	-0.305	Moderate	0.219	-0.781	0.219
TENA_Letter_Time	-0.645	Strong	0.002*	-0.870	-0.208
TENA_Letter_Error	-0.804	Strong	< .001*	-0.924	-0.555
TENA_Num_Time	-0.452	Moderate	0.046	-0.782	0.033
TENA_Num_Error	-0.454	Moderate	0.067	-0.816	0.069

* significant;

Variable control: CMMS (percentile);

Confidence interval (CI) based on bootstrap (1000 replications)

Furthermore, as shown in Table 3, linear regression analysis for TRPP (phonological working memory) indicated that the model explained 25% of the variance (R^2^ = 0.25, f = 0.33, medium effect) with statistical significance (F(1.23) = 7.663, p = 0.011). The regression coefficient for SPRouT (β = −2.044, p = 0.011) indicated a negative relationship, suggesting that higher scores on SPRouT are associated with lower scores on TRPP. The diagnoses showed statistical adequacy, with no collinearity problems (Condition Index = 3.864) and residuals distributed in an approximately random and normal manner, although discrepancies at the extremes suggest the need to pay attention to possible outliers.

**Table 3:** Linear regression analysis (independent variable: SPRouT)

**Model**	**R^2^**	***f* ^2^**	**F (df)**	***p***	**β**	**95% CI (β)**
TRPP	0.25	0.33	F(1.23)=7.663	0.011*	-2.044	[-3.571. -0.516]
PCFO	0.314	0.46	F(1.24)=11.003	0.003*	-3.853	[-6.251. -1.456]
TENA_color	0.235	0.31	F(1.23)=7.066	0.014*	-2.286	[-4.065. -0.507]
TENA_obj	0.03	0.03	F(1.23)=0.702	0.411	-0.919	[-3.187. 1.349]
TENA_letter	0.328	0.49	F(1.19)=9.259	0.007*	-3.048	[-5.145. -0.952]
TENA_num	0.155	0.18	F(1.19)=3.477	0.078	-2.19	[-4.648. 0.268]

* significant

Regarding PCFO (phonological awareness), the model explained 31.4% of the variance (R^2^ = 0.314, f = 0.46, large effect), with statistical significance (F(1.24) = 11.003, p = 0.003). The SPRouT coefficient (β = −3.853, p = 0.003) also indicated a negative relationship. The analysis of the residuals reinforced the adequacy of the model, with an approximately random and normal distribution. Small deviations at the extremes, attributed to outliers, indicate the need for greater attention in future analyses.

In TENA Color (time), the model explained 23.5% of the variance (R^2^ = 0.235, f = 0.31, medium effect), statistically significant (F(1.23) = 7.066, p = 0.014). The SPRouT coefficient (β = −2.286, p = 0.014) indicated a negative relationship, with a robust confidence interval (-4.065 to -0.507). No collinearity problems were observed (Condition Index = 3.846), and the model presented a moderate standard error of estimate (RMSE = 23.401).

For TENA Object (time), the model explained only 3.0% of the variance (R^2^ = 0.030, f = 0.03, small effect) and was not statistically significant (F(1.23) = 0.702, p = 0.411). The SPRouT coefficient (β = −0.919, p = 0.411) indicated a negative relationship, but without statistical significance, with a confidence interval encompassing zero (-3.187 to 1.349). In addition, the model showed high data dispersion (RMSE = 29.841).

In TENA Letter (time), the model explained 32.8% of the variance (R^2^ = 0.328, f = 0.49, large effect), with statistical significance (F(1.19) = 9.259, p = 0.007). The SPRouT coefficient (β = −3.048, p = 0.007) indicated a negative relationship, with a robust confidence interval (-5.145 to -0.952). The model diagnostics did not reveal collinearity problems (Condition Index = 3.609), and the standard error of estimate was moderate (RMSE = 25.458).

Finally, in TENA Number (time), the model explained 15.5% of the variance (R^2^ = 0.155, f = 0.18, medium effect), but it was not statistically significant (F(1.19) = 3.477, p = 0.078). The SPRouT coefficient (β = −2.190, p = 0.078) indicated a negative relationship, but without statistical significance, with a confidence interval encompassing zero (-4.648 to 0.268). The model also exhibited a high standard error (RMSE = 30.547).

## DISCUSSION

This research aimed to investigate preliminary validity evidence for the Brazilian version of the SPRouT^(16)^, considering its relationship with standardized measures of phonological skills associated with reading development. Specifically, it analyzed both the convergent validity and the predictive potential of the SPRouT, controlling for the general intelligence level.

Previous research has already demonstrated that phonological processing skills are precursors to reading skills, being fundamental for the early identification of the risk of literacy difficulties^(23,24)^. In this context, the results obtained reinforce the relevance of the SPRouT as a promising instrument for early screening, by showing significant correlations between its score and performance in tasks of phonological awareness, phonological memory, and rapid automatic naming. Considering that factor analyses have not yet been carried out with the SPRouT-D, and based on the one-dimensionality observed in a national study with a Brazilian screening instrument^(16)^, it was decided to use the total SPRouT score as a risk indicator.

Correlation analyses showed statistically significant negative relationships between total SPRouT scores and PCFO, TRPP, and TENA (Color and Letter). This indicates that the higher the SPRouT score (higher risk), the worse the performance in the assessed phonological skills, which supports convergent validity. The strongest association was observed between SPRouT and PCFO, corroborating the literature that highlights phonological awareness as one of the most essential skills for early detection of reading difficulties^(25-27)^.

From a statistical point of view, correlations were performed controlling for the effect of general intelligence (percentile obtained in the CMMS) to isolate the specific contribution of phonological skills on SPRouT scores. This methodological choice is justified because intelligence, although not determinant, can influence performance in phonological processing tasks^(28,29)^. By controlling for this variable, the aim was to reduce the bias associated with differences in overall intellectual functioning, allowing for a more precise analysis of the validity of the SPRouT based on the skills it aims to assess.

In addition, regression analyses demonstrated that the SPRouT explains 23% to 32% of the variance in phonological processing tasks, with medium to large effects. The greatest explanatory power was found in the model with the PCFO, followed by TENA – Letter, TRPP, and TENA – Color. These findings support not only convergent validity but also the predictive potential of the SPRouT, even in a cross-sectional study. Residual and collinearity analyses showed statistical adequacy of the models, although the presence of possible outliers at the extremes indicates the need for caution and refinement in future studies.

On the other hand, the models for TENA (object and number) did not show statistical significance, indicating that SPRouT may not be associated with all types of rapid automatic naming tasks, especially those with less direct relation to initial reading. The literature indicates that rapid automatic naming of alphanumeric stimuli tends to correlate more strongly with spelling and reading than tasks with colors and objects^(30)^.

From a psychometric point of view, the findings are aligned with the Standards for Educational and Psychological Testing^(13)^, which highlight the importance of gathering validity evidence based on the relationship with external variables, especially in the initial stages of validating an instrument. By showing sensitivity to phonological skills that predict reading, the SPRouT can be a useful screening instrument.

Furthermore, although the SPRouT was originally developed for pediatric professionals in the North American context^(15)^, its cross-cultural adaptation to Brazilian Portuguese^(16)^ can broaden its possibilities of use. It is believed that different professionals who work with child development and learning (e.g., psychologists, speech-language-hearing pathologists, educational psychologists, and educators) can incorporate the instrument in clinical, school, and interdisciplinary contexts.

Despite the promising findings, the study has limitations that should be mentioned. The sample was small and restricted to a single private school in Northeastern Brazil, which limits the generalization of the results to other regions, school realities, and socioeconomic contexts. Moreover, data collection at school was chosen for convenience, which facilitated access and standardized application, but does not reflect the original clinical context for which the SPRouT was developed. Although the instrument was designed for use in clinical settings, especially by pediatricians, this study used a school sample, which represents a limitation in terms of suitability to the original target audience and direct practical applicability.

Therefore, it is recommended that future research include clinical samples and healthcare settings to broaden the evidence of the instrument's validity in different application environments. Finally, the sample included children with and without suspected learning difficulties, without formal diagnostic confirmation, introducing uncontrolled nosological variation. In addition, some children did not complete all instruments due to attentional difficulties or low cooperation, reducing the number of valid responses in some analyses.

## CONCLUSION

Despite its exploratory nature, the study provided initial evidence of convergent validity for the Brazilian version of the SPRouT, considering its relationships with constructs related to reading development. Additionally, the results indicate its predictive potential, although longitudinal studies with clinical samples are needed to confirm this property. Further investigation of other sources of validity and reliability evidence (e.g., internal consistency and test-retest) using larger and more diverse samples is recommended.
