# Supplementary material for: Preliminary validation of the Brazilian version of SPRouT for screening reading difficulties
Source: Codas. 2026 Jul 8;38(5):e20250165. doi: 10.1590/2317-1782/e20250165en (PMC13440452; doi:10.1590/2317-1782/e20250165en)
Supplement: Translation

# Translation: Validação preliminar da versão brasileira do SPRouT para triagem de dificuldades de leitura

**DOI:** 10.1590/2317-1782/e20250165pt

**Authors:** Beatriz de Souza dos Reis França, Lucas Alves Maia, Ricardo Franco de Lima, Cíntia Alves Salgado-Azoni

**Affiliations:** 1 Programa Associado de Pós-graduação em Fonoaudiologia UFRN/UNCISAL/UFPB – PPgFon, Universidade Federal do Rio Grande do Norte – UFRN - Natal (RN), Brasil.; 2 Universidade Federal do Rio Grande do Norte – UFRN - Natal (RN), Brasil.; 3 Programa de Pós-graduação em Psicologia, Universidade São Francisco – USF - Campinas (SP), Brasil.

**Keywords:** Triagem, Leitura, Dislexia, Alfabetização, Psicometria

## Abstract

**Objetivo:**

Investigar evidências preliminares de validade baseada na relação com variáveis externas da versão brasileira do Screening Pediatric Patients for Reading Difficulties Test (SPRouT).

**Método:**

Após aprovação pelo Comitê de Ética, 27 crianças de quatro a oito anos foram avaliadas individualmente com o SPRouT e com instrumentos padronizados que medem inteligência e habilidades de processamento fonológico. As análises incluíram correlações de Spearman, controlando o efeito da inteligência, e regressões lineares simples para verificar a capacidade preditiva do SPRouT.

**Resultados:**

Foram observadas correlações negativas moderadas a fortes entre os escores do SPRouT e medidas de consciência fonológica, memória de trabalho fonológica e nomeação automática rápida. O escore total do SPRouT explicou de 23% a 32% da variância nas medidas fonológicas, com efeitos de magnitude média a alta.

**Conclusão:**

Os achados fornecem evidências iniciais de validade convergente para o SPRouT, ao demonstrar associações com habilidades relacionadas ao desenvolvimento da leitura. As análises de regressão sugerem seu potencial preditivo, embora estudos longitudinais e com amostras clínicas sejam necessários para confirmar essa propriedade. O SPRouT representa uma ferramenta promissora para a triagem de dificuldades de leitura, recomendando-se a ampliação das investigações psicométricas com amostras maiores e mais diversificadas.

## INTRODUÇÃO

A aquisição da leitura é um marco essencial no desenvolvimento infantil, fundamental para o sucesso acadêmico e para a plena participação social. Antes do início do ensino formal, o desenvolvimento de habilidades precursoras da leitura é indispensável, pois elas estabelecem alicerces para a proficiência leitora subsequente^(1,2)^.

Diversos estudos têm investigado os componentes do processamento fonológico, evidenciando suas contribuições para a aquisição e o desenvolvimento da leitura^(3,4)^. Entre esses componentes, a consciência fonológica se destaca como um forte preditor do desempenho de leitura, considerada uma importante competência a ser incluída em instrumentos de triagem para dificuldades de leitura. Essa habilidade permite que as crianças distingam e manipulem os sons da fala, facilitando, consequentemente, o processo de leitura^(5)^.

A nomeação automática rápida, frequentemente medida pela velocidade de nomeação de estímulos visuais familiares, por sua vez, tem se mostrado um marcador estável ao longo do desenvolvimento, com forte capacidade preditiva de leitura^(6)^. Além disso, a memória fonológica, usualmente avaliada por tarefas com dígitos ou pseudopalavras, também tem demonstrado relação significativa com a leitura inicial, atuando como preditora do sucesso precoce na aprendizagem da leitura^(7)^.

Uma revisão sistemática conduzida por Schoenel et al.^(8)^ apontou que crianças com dificuldades no processo de alfabetização apresentam desempenho inferior em diversas habilidades do processamento fonológico, quando comparadas àquelas com alfabetização adequada. Esse dado reforça a importância do processamento fonológico como um preditor chave para o desenvolvimento da leitura e da escrita.

Todavia, apesar do conhecimento dessas habilidades e do impacto da leitura no desenvolvimento, os desafios associados às dificuldades de leitura e sua avaliação precoce permanecem expressivos. A identificação tardia de dificuldades de leitura pode gerar repercussões significativas no desempenho acadêmico e no desenvolvimento socioemocional da criança, como desmotivação, baixa autoestima e dificuldades de aprendizagem, os quais podem persistir ao longo da vida adulta^(9)^.

No Brasil, os resultados do *Sistema de Avaliação da Educação Básica* (Saeb 2021) evidenciam desafios persistentes na proficiência em Língua Portuguesa entre estudantes do 2º ano do Ensino Fundamental. Mais da metade dos alunos avaliados foi classificada nos níveis mais baixos da escala de proficiência que avalia o domínio do princípio alfabético, habilidades de leitura e produção textual^(10)^. Esse cenário evidencia a necessidade de aprimorar a identificação de crianças de risco para o desenvolvimento da alfabetização, assim como intervenções eficazes.

No ambiente clínico, os pediatras podem desempenhar um papel importante no rastreamento de dificuldades de leitura, mediante análise do histórico familiar e uso de ferramentas de triagem validadas e normatizadas^(11)^. Embora a identificação precoce enfrente desafios quanto à precisão, sua aplicação permite intervenções antecipadas, que podem prevenir ou atenuar dificuldades de leitura antes que se consolidem^(12)^.

Para que instrumentos de triagem sejam utilizados com rigor científico e aplicabilidade clínica, é essencial que suas propriedades psicométricas sejam sistematicamente investigadas. Conforme estabelecido pelos *Standards for Educational and Psychological Testing^(^* ^13)^, a validade de um instrumento deve ser sustentada por múltiplas fontes de evidência, a saber: (1) evidência baseada no conteúdo, (2) na estrutura interna, (3) na relação com variáveis externas, (4) nos processos de resposta e (5) nas consequências do uso do teste.

Dentre essas, a evidência baseada em relações com variáveis externas refere-se ao grau em que os escores de um teste se associam a outras medidas ou desfechos, de modo consistente com o construto que se pretende avaliar^(13)^. Esse tipo de evidência é particularmente relevante em estudos iniciais de validação, pois permite verificar a coerência teórica e a utilidade prática do instrumento ao se correlacionar com medidas estabelecidas.

Apesar da importância desse tipo de evidência, observa-se, no contexto brasileiro, uma escassez de estudos de validação para medidas de triagem e avaliação do desenvolvimento infantil^(14)^. Essa limitação indica não apenas a necessidade de maior rigor na seleção e utilização desses instrumentos, mas também reforça a importância de fomentar pesquisas voltadas à validação destes, com vistas à disponibilização de ferramentas mais abrangentes e confiáveis para a identificação de riscos ao desenvolvimento.

Assim, o instrumento de triagem *Screening Pediatric Patients for Reading Difficulties Test* (SPRouT) foi originalmente desenvolvido com base em uma perspectiva clínica, com o objetivo de atender às demandas práticas e à rotina dinâmica dos médicos pediatras. Trata-se de uma breve lista de verificação, ainda não validada, para avaliar dificuldades de leitura em crianças de quatro a oito anos^(15)^. Sua tradução e adaptação transcultural já foram exploradas tanto para o português brasileiro, sob o título *Brazilian Version of the Screening Pediatric Patients for Reading Difficulties Test^(^* ^16)^, quanto para o espanhol, com a versão *Prueba pediátrica de detección de dificultades de alfabetización: Borrador* - PEDAL-B^(17)^. Contudo, ainda são necessários estudos que investiguem suas propriedades psicométricas.

Considerando os aspectos mencionados, o presente estudo objetivou investigar evidências preliminares de validade do SPRouT a partir da relação com outras variáveis, especificamente por meio da associação entre seus escores e medidas de habilidades fonológicas reconhecidamente associadas ao desenvolvimento da leitura.

## MÉTODO

### Tipo de estudo e aspectos éticos

Estudo metodológico transversal descritivo e analítico, que consiste em uma etapa preliminar de validação do instrumento de triagem *Brazilian Version of the Screening Pediatric Patients for Reading Difficulties Test* (SPRouT), a partir das etapas de construção, tradução e adaptação transcultural já concluídas^(16)^.

Este estudo foi aprovado pelo Comitê de Ética em Pesquisa (CEP) da Universidade São Francisco (USF), sob parecer número 6.042.270, de acordo com a resolução do Conselho Nacional de Saúde - CNS 466/12 sobre Diretrizes e Normas Regulamentadoras de Pesquisas Envolvendo Seres Humanos. Todos os participantes assinaram o Termo de Consentimento Livre e Esclarecido (TCLE).

### Participantes

Para este estudo, crianças de quatro a oito anos foram incluídas, falantes de português, matriculadas no ensino regular privado, com ou sem queixas relacionadas à linguagem oral e/ou escrita, conforme relato dos pais ou responsáveis legais. A inclusão de crianças com queixas foi justificada pelo maior risco de apresentarem transtornos de aprendizagem. O critério de exclusão estabelecido foi a desistência dos participantes durante o processo de avaliação.

O estudo contou com a participação de 27 crianças e seus responsáveis, com idades entre 52 e 106 meses (Média = 76,89; DP = 16,05), de ambos os sexos, sendo 13 meninos (48%) e 14 meninas (52%). Quanto à escolaridade, os participantes estavam distribuídos entre nível 4 (n = 7; 26%) e nível 5 da educação infantil (n = 5; 19%), 1º ano (n = 9; 33%), 2º ano (n = 3; 11%) e 3º ano do ensino fundamental I (n = 3; 11%). Todas as crianças frequentavam uma escola particular de uma cidade do Nordeste brasileiro.

No grupo total, 19 crianças (70%) não apresentavam diagnósticos, enquanto 8 crianças (30%) possuíam hipóteses diagnósticas específicas, conforme relato dos pais. Dentre essas, destacaram-se condições como Transtorno do Espectro Autista (TEA), Apraxia de Fala na Infância (AFI), transtorno fonológico e Transtorno de Déficit de Atenção/Hiperatividade (TDAH).

Embora o SPRouT tenha sido desenvolvido para uso clínico em pacientes pediátricos, sua aplicação em ambiente educacional foi priorizada devido à disponibilidade da amostra e dos pesquisadores. Essa escolha metodológica pode fornecer contribuições para seu uso em diferentes contextos, porém representa uma limitação quanto à adequação ao público-alvo original.

### Materiais

Do ponto de vista psicométrico, para atender aos objetivos do estudo, foram selecionados instrumentos amplamente utilizados em contextos clínicos e educacionais, todos normatizados para a população brasileira e com evidências de validade. Esses instrumentos avaliam diferentes componentes do processamento fonológico, além da inteligência.

#### Versão brasileira do Teste de Triagem para Dificuldades de Leitura em Pacientes Pediátricos (SPRouT)^(16)^

O instrumento consiste em cinco formulários (A-E), selecionados com base na idade da criança. Cada formulário é composto por duas seções: uma com questões referentes à história clínica e de desenvolvimento da criança para ser realizada com os pais e outra com itens destinados à realização de uma avaliação clínica rápida com a criança. A seção clínica pode conter de cinco a oito itens, variando conforme o formulário utilizado. As tarefas desta seção avaliam habilidades linguísticas consideradas pré-requisitos para a leitura, como a consciência fonológica e a memória de trabalho fonológica^(16)^. Para fins de avaliação, são atribuídas pontuações aos itens que indicam erros ou sinais de possíveis dificuldades no desenvolvimento, na fala e na aprendizagem. No presente estudo, foi utilizado o escore bruto total, que representa a soma desses indicadores. Assim, quanto maior o escore, maior o risco de a criança apresentar dificuldades de leitura.

#### Prova de Consciência Fonológica por produção Oral - PCFO^(18)^

Esta prova avalia a habilidade de consciência fonológica, sendo composta por 10 subtestes: síntese silábica e fonêmica, julgamento de rimas e de aliterações, segmentação, manipulação, e transposição de sílabas e fonemas. Cada subteste possui, além da instrução, dois itens de treino e quatro itens de teste. Atribui-se um ponto para cada resposta correta. A PCFO deve ser aplicada individualmente e o tempo médio de aplicação é de 20 minutos. O escore bruto de todos os subtestes, que corresponde ao total de acertos, é convertido em uma pontuação-padrão para fins de classificação. Estudos de fidedignidade identificaram índices que variaram de 0,86 a 0,91 para a PCFO, dependendo do método utilizado (consistência interna e método das metades), em amostras de estudantes da educação infantil e do ensino fundamental^(18)^.

#### Teste de Repetição de Palavras e Pseudopalavras - TRPP^(19)^

Este teste avalia a memória de curto prazo fonológica por meio de uma tarefa de repetição de palavras e pseudopalavras. Nesta prova, o aplicador pronuncia para a criança uma sequência de duas a seis palavras, com intervalo de 1 segundo entre elas, sendo a tarefa da criança repetir as palavras na mesma sequência. Há duas sequências para cada comprimento (palavras e pseudopalavras). O teste é aplicado de forma individual, sendo atribuído 1 ponto para cada sequência correta, que se obtém em uma pontuação-padrão para a classificação. O tempo médio de aplicação é de 7 minutos. Quanto à fidedignidade, estudos identificaram índices variando de 0,77 a 0,83 para o TRPP, considerando os métodos de consistência interna e das metades. Além disso, o método de teste-reteste indicou um índice de correlação de 0,73^(19)^.

#### Teste de Nomeação Automática - TENA^(20)^

O teste avalia individualmente a habilidade de nomeação automática rápida, sendo composto por quatro subtestes: nomeação de cores, objetos, letras e números. Cada subteste contém cinco estímulos, que são repetidos de forma randomizada dez vezes em cada uma das cinco linhas, totalizando 50 estímulos por prancha. O examinador solicita que o avaliado nomeie cada estímulo o mais rápido possível e sem cometer erros, tanto na prancha de treino quanto na de teste. Durante a avaliação, são registrados o número de erros e o tempo total de nomeação em cada subteste^(20)^. Por se tratar de um instrumento normatizado para a população brasileira, foram considerados os percentis de tempo e de erros para cada subteste. Estudos de fidedignidade, com base na estabilidade temporal, demonstraram correlações positivas, significativas e com magnitude variando de moderada a alta entre as aplicações, sobretudo nos escores de tempo.

#### Escala de Maturidade Mental Colúmbia - EMMC^(21)^

Trata-se de um instrumento destinado à avaliação da inteligência fluida (*Gf)*, que não requer respostas orais, é de uso restrito ao profissional da Psicologia e possui aprovação do Sistema de Avaliação de Testes Psicológicos (SATEPSI). É composto por 92 itens de classificação pictóricos e figurativos, organizados em oito níveis, conforme a faixa etária da criança. No presente estudo, foi utilizado o escore total de acertos, expresso em percentil. Estudos brasileiros relatam índices de precisão da EMMC variando de 0,86 a 0,93.

### Procedimentos

#### Recrutamento

O processo de recrutamento dos participantes foi realizado com o auxílio da secretaria escolar, que colaborou na identificação dos alunos ativos a partir do banco de dados institucional. Inicialmente, foi realizado contato telefônico com os responsáveis pelos alunos que se encontravam em faixa etária compatível com os critérios de inclusão do estudo.

Dentre as principais razões para a recusa dos responsáveis, destacou-se a ocorrência de acompanhamento terapêutico em andamento com a criança. Os responsáveis que demonstraram interesse em participar do estudo foram encaminhados, por meio digital, a um formulário contendo informações detalhadas sobre a história clínica da criança, juntamente com o Termo de Consentimento Livre e Esclarecido (TCLE). Além disso, foi enviado um material informativo complementar, desenvolvido pelos autores utilizando a plataforma *Canva*, no formato PDF, por meio do aplicativo de mensagens *WhatsApp Business*. A Figura 1 apresenta um fluxograma do processo de recrutamento, desde o contato inicial até a seleção da amostra final.

**Figura 1:** Fluxograma do processo de recrutamento dos participantes

#### Avaliação

A coleta de dados foi conduzida por conveniência em duas unidades de uma instituição de ensino particular localizada em uma cidade no nordeste do Brasil, selecionadas em razão da viabilidade de acesso por parte dos pesquisadores. O processo ocorreu durante os meses de julho a agosto de 2024. A primeira unidade era composta por alunos da educação infantil (nível 4 e 5) até o primeiro ano do ensino fundamental I, enquanto a segunda era destinada a estudantes do segundo ano do ensino fundamental I até o ensino médio.

As avaliações ocorreram nas unidades escolares durante o horário de aula, previamente acordado com a equipe pedagógica para não gerar prejuízos no desempenho acadêmico dos alunos. A aplicação dos instrumentos foi conduzida por uma equipe interdisciplinar calibrada previamente ao início da coleta, composta por quatro fonoaudiólogos, um aluno de graduação em fonoaudiologia, uma psicóloga e uma graduanda em psicologia, em salas com condições acústicas e visuais adequadas para garantir a concentração e o conforto dos participantes.

Devido às limitações de espaço físico, os grupos foram organizados com, no máximo, quatro crianças por sala, sendo cada uma acompanhada por um pesquisador. As avaliações duraram, em média, 60 minutos por criança. Em alguns casos, foi necessário dividir o tempo em duas sessões, em razão da exigência de que a EMMC fosse aplicada por um profissional da psicologia. Além disso, nem todas as crianças conseguiram completar todos os instrumentos, em função de dificuldades atencionais ou falta de colaboração durante a coleta.

#### Análise de dados

Análises descritivas foram realizadas para caracterizar a amostra quanto às variáveis sociodemográficas, incluindo idade, sexo e nível de escolaridade. As estatísticas descritivas (média, desvio padrão, valores mínimo e máximo) foram calculadas para as variáveis contínuas, enquanto frequências absolutas e percentuais foram reportadas para as variáveis categóricas. Ressalta-se que houve variação no número de participantes incluídos na análise estatística, em razão de respostas incompletas ou inválidas. Essas variações foram decorrentes de dificuldades de compreensão ou colaboração por parte de algumas crianças durante a coleta de dados.

Considerando que os escores dos testes não tiveram distribuição normal (Shapiro-Wilk; p<0,05) foi conduzida análise de correlação de *Spearman* para investigar as relações entre os escores da pontuação total do SPRouT e os instrumentos que avaliam processamento fonológico. Como variações no nível de inteligência podem influenciar o desempenho em tarefas fonológicas, optou-se por controlar esse fator nas análises. Assim, o percentil da EMMC foi utilizado como variável de controle, com o objetivo de isolar o efeito da inteligência geral e verificar de forma mais precisa a relação entre o SPRouT e as habilidades fonológicas avaliadas. As correlações foram interpretadas de acordo com os critérios de magnitude propostos por Cohen^(22)^, classificadas como: entre 0,10 e 0,29 (fraca); entre 0,30 e 0,49 (moderada); e entre 0,50 e 1 (fortes).

Uma análise de regressão linear foi realizada para avaliar o potencial preditivo do escore total do SPRouT em relação aos percentis dos testes que avaliam processamento fonológico. Assim, o escore total do SPRouT foi considerado como variável independente, enquanto os percentis dos instrumentos (PCFO, TRPP e TENA) foram definidos como variáveis dependentes. Os critérios para avaliação do modelo incluíram: coeficiente de determinação (R^2^): para medir a proporção de variância explicada pela variável independente; significância estatística (p): considerou-se significativo p<0,05; tamanho do efeito (*f ^2^* ): calculado pela fórmula *f* ^2^=R^2^/(1−R^2^), interpretado como: pequeno: *f* ^2^≥0,02, médio: *f* ^2^≥0,15 e grande: *f* ^2^≥0,35.

## RESULTADOS

A Tabela 1 apresenta a análise descritiva dos instrumentos utilizados. As classificações da EMMC foram distribuídas da seguinte forma: extremamente baixo (n = 1; 4%), muito baixo (n = 1; 4%), média (n = 19; 70%) e média superior (n = 6; 22%).

**Tabela 1:** Estatística descritiva dos escores dos instrumentos administrados

**Escores**		**N**		**M**		**DP**		**Min.**		**Max.**	
SPRouT (história)		26		3,73		1,95		1,00		8,00	
SPRouT (clínico)		26		6,15		4,49		0,00		16,00	
SPRouT (total)		26		10,00		5,58		2,00		24,00	
TRPP (palavras)		26		104,92		21,12		75,00		145,00	
TRPP (pseudopalavras)		24		106,63		23,19		82,00		149,00	
TRPP (total)		26		104,89		22,16		69,00		155,00	
PCFO (total)		27		110,41		37,78		61,00		211,00	
TENA Cor (tempo)		26		44,81		26,63		10,00		90,00	
TENA Cor (erro)		22		39,32		27,53		10,00		90,00	
TENA Obj (tempo)		26		46,54		29,39		10,00		90,00	
TENA Obj (erro)		20		33,50		25,86		10,00		90,00	
TENA Letra (tempo)		22		44,77		29,86		10,00		90,00	
TENA Letra (erro)		19		35,53		27,43		10,00		90,00	
TENA Num (tempo)		22		42,27		31,84		10,00		90,00	
TENA Num (erro)		19		28,68		25,92		10,00		90,00	
EMMC		27		57,96		21,97		1,00		90,00	

**Legenda:** N = amostra; M = Média; DP = desvio padrão; Min.= Mínimo; Máx.= Máximo

Conforme a Tabela 2, os resultados indicaram correlações negativas significativas entre o SPRouT e a maioria dos instrumentos (subteste de pseudopalavras do TRPP, escore total do TRPP, PCFO, TENA cor - tempo, e TENA letra - tempo e erro). As correlações variaram de fraca *(rho* = -0.174) a forte (*rho* = -0.804).

**Tabela 2:** Correlação de Spearman entre o SPRouT e os demais instrumentos

	**Coeficiente de correlação de Spearman**	**Magnitude**	**p**	**IC 95% - Limite inferior**	**IC 95% - Limite superior**
TRPP_Rep_palavras	-0,326	Moderada	0,120	-0,674	0,089
TRPP_Rep_pseudo	-0,484	Moderada	0,022*	-0,777	-0,096
TRPP	-0,524	Forte	0,009*	-0,796	-0,183
PCFO	-0,719	Forte	< ,001*	-0,889	-0,408
TENA_Cor_Tempo	-0,494	Moderada	0,014*	-0,770	-0,119
TENA_Cor_Erro	-0,330	Moderada	0,155	-0,674	0,111
TENA_Obj_Tempo	-0,174	Fraca	0,417	-0,533	0,211
TENA_Obj_Erro	-0,305	Moderada	0,219	-0,781	0,219
TENA_Letra_Tempo	-0,645	Forte	0,002*	-0,870	-0,208
TENA_Letra_Erro	-0,804	Forte	< ,001*	-0,924	-0,555
TENA_Num_Tempo	-0,452	Moderada	0,046	-0,782	0,033
TENA_Num_Erro	-0,454	Moderada	0,067	-0,816	0,069

* significativo;

Controle de variável: EMMC (percentil);

Intervalo de confiança (IC) baseado em bootstrap (1000 replicações)

Ademais, conforme mostra a Tabela 3, a análise de regressão linear para o TRPP (memória fonológica de trabalho) indicou que o modelo explicou 25% da variância (R^2^ = 0,25, *f* = 0,33, efeito médio) com significância estatística (F(1,23) = 7,663, p = 0,011). O coeficiente de regressão do SPRouT (β = −2,044, p = 0,011) indicou uma relação negativa, sugerindo que escores mais altos no SPRouT estão associados a escores mais baixos no TRPP. Os diagnósticos mostraram adequação estatística, sem problemas de colinearidade (*Condition Index* = 3,864) e resíduos distribuídos de forma aproximadamente aleatória e normal, embora discrepâncias nos extremos sugiram a necessidade de atenção a possíveis *outliers*.

**Tabela 3:** Análise de regressão linear (variável independente: SPRouT)

**Modelo**	**R^2^**	***f* ^2^**	**F (df)**	***p***	**β**	**IC 95% (β)**
TRPP	0,25	0,33	F(1,23)=7,663	0,011*	-2,044	[-3,571, -0,516]
PCFO	0,314	0,46	F(1,24)=11,003	0,003*	-3,853	[-6,251, -1,456]
TENA_cor	0,235	0,31	F(1,23)=7,066	0,014*	-2,286	[-4,065, -0,507]
TENA_obj	0,03	0,03	F(1,23)=0,702	0,411	-0,919	[-3,187, 1,349]
TENA_letra	0,328	0,49	F(1,19)=9,259	0,007*	-3,048	[-5,145, -0,952]
TENA_num	0,155	0,18	F(1,19)=3,477	0,078	-2,19	[-4,648, 0,268]

* significativo

Em relação à PCFO (Consciência Fonológica), o modelo explicou 31,4% da variância (R^2^ = 0,314, *f* = 0,46, efeito grande), com significância estatística (F(1,24) = 11,003, p = 0,003). O coeficiente do SPRouT (β = −3,853, p = 0,003) também indicou uma relação negativa. A análise dos resíduos reforçou a adequação do modelo, com distribuição aproximadamente aleatória e normal. Pequenos desvios nos extremos, atribuídos a valores atípicos, indicam a necessidade de maior atenção em análises futuras.

No TENA Cor (tempo), o modelo explicou 23,5% da variância (R^2^ = 0,235, *f* = 0,31, efeito médio), estatisticamente significativo (F(1,23) = 7,066, p = 0,014). O coeficiente do SPRouT (β = −2,286, p = 0,014) indicou uma relação negativa, com um intervalo de confiança robusto (-4,065 a -0,507). Não foram observados problemas de colinearidade (*Condition Index*=3,846) e o modelo apresentou um erro padrão de estimativa moderado (RMSE = 23,401).

Já para o TENA Objeto (tempo), o modelo explicou apenas 3,0% da variância (R^2^ = 0,030, *f* = 0,03, efeito pequeno) e não foi estatisticamente significativo (F(1,23) = 0,702, p = 0,411). O coeficiente do SPRouT (β = −0,919, p = 0,411) indicou uma relação negativa, mas sem significância estatística, com intervalo de confiança abrangendo zero (-3,187 a 1,349). Além disso, o modelo apresentou dispersão elevada nos dados (RMSE = 29,841).

No TENA Letra (tempo), o modelo explicou 32,8% da variância (R^2^ = 0,328, *f* = 0,49, efeito grande), com significância estatística (F(1,19) = 9,259, p = 0,007). O coeficiente do SPRouT (β = −3,048, p = 0,007) indicou uma relação negativa, com um intervalo de confiança robusto (-5,145 a -0,952). Os diagnósticos do modelo não revelaram problemas de colinearidade (*Condition Index*=3,609) e o erro padrão de estimativa foi moderado (RMSE=25,458).

Por fim, no TENA número (tempo), o modelo explicou 15,5% da variância (R^2^ = 0,155, *f* = 0,18, efeito médio), mas não foi estatisticamente significativo (F(1,19) = 3,477, p = 0,078). O coeficiente do SPRouT (β = −2,190, p = 0,078) indicou uma relação negativa, mas sem significância estatística, com intervalo de confiança abrangendo zero (-4,648 a 0,268). O modelo também apresentou um erro padrão elevado (RMSE = 30,547).

## DISCUSSÃO

A presente pesquisa teve como objetivo investigar evidências preliminares de validade da versão brasileira do SPRouT^(16)^, considerando sua relação com medidas padronizadas de habilidades fonológicas associadas ao desenvolvimento da leitura. Especificamente, foram analisadas tanto a validade convergente quanto o potencial preditivo do SPRouT, com controle do nível de inteligência geral.

Pesquisas anteriores já demonstraram que as habilidades de processamento fonológico são precursoras das competências de leitura, sendo fundamentais para a identificação precoce do risco de dificuldades na alfabetização^(23,24)^. Nesse contexto, os resultados obtidos reforçam a relevância do SPRouT como um instrumento promissor para triagem precoce, ao evidenciar correlações significativas entre seu escore e o desempenho em tarefas de consciência fonológica, memória fonológica e nomeação automática rápida. Considerando que ainda não foram realizadas análises fatoriais com o SPRouT-D, e com base na unidimensionalidade observada em estudo nacional com instrumento brasileiro de triagem^(16)^, optou-se por utilizar o escore total do SPRouT como indicador de risco.

As análises de correlação mostraram relações negativas e estatisticamente significativas entre os escores totais do SPRouT e os testes PCFO, TRPP, TENA (Cor e Letra). Isso indica que quanto maior a pontuação no SPRouT (maior risco), pior o desempenho nas habilidades fonológicas avaliadas, o que sustenta a validade convergente. A associação mais forte foi observada entre o SPRouT e a PCFO, corroborando a literatura que destaca a consciência fonológica como uma das habilidades mais essenciais para detecção precoce de dificuldades de leitura^(25-27)^.

Do ponto de vista estatístico, as correlações foram realizadas controlando o efeito da inteligência geral (percentil obtido na EMMC), a fim de isolar a contribuição específica das habilidades fonológicas sobre os escores do SPRouT. Essa escolha metodológica se justifica porque a inteligência, apesar de não determinante, pode influenciar o desempenho em tarefas de processamento fonológico^(28,29)^. Ao controlar essa variável, buscou-se reduzir o viés associado a diferenças no funcionamento intelectual geral, permitindo uma análise mais precisa da validade do SPRouT com base nas habilidades que ele realmente se propõe a avaliar.

Além disso, as análises de regressão demonstraram que o SPRouT explica de 23% a 32% da variância em tarefas de processamento fonológico, com efeitos médios a grandes. O maior poder explicativo foi encontrado no modelo com o PCFO, seguido pelo TENA – Letra, TRPP e TENA – Cor. Esses achados sustentam não apenas a validade convergente, mas também o potencial preditivo do SPRouT, mesmo em um estudo transversal. As análises de resíduos e colinearidade mostraram adequação estatística dos modelos, ainda que a presença de possíveis *outliers* nos extremos indique a necessidade de cautela e refinamento em estudos futuros.

Por outro lado, os modelos para TENA (objeto e número) não apresentaram significância estatística, indicando que o SPRouT pode não estar associado a todos os tipos de tarefas de nomeação automática rápida, especialmente aquelas com menor relação direta com a leitura inicial. A literatura indica que a nomeação automática rápida de estímulos alfanuméricos tende a se correlacionar mais fortemente com a ortografia e a leitura do que tarefas com cores e objetos^(30)^.

Do ponto de vista psicométrico, os achados estão alinhados com os *Standards for Educational and Psychological Testing^(^* ^13)^, que destacam a importância de reunir evidências de validade com base na relação com variáveis externas, sobretudo nas etapas iniciais de validação de um instrumento. Ao se mostrar sensível às habilidades fonológicas preditoras da leitura, o SPRouT pode ser um instrumento útil para fins de triagem.

Além disso, é importante destacar que, embora o SPRouT tenha sido originalmente desenvolvido para profissionais da pediatria no contexto norte-americano^(15)^, sua adaptação transcultural para o português brasileiro^(16)^ pode ampliar suas possibilidades de uso. Considera-se que o instrumento possa ser incorporado por diferentes profissionais que atuam com desenvolvimento infantil e aprendizagem, como psicólogos, fonoaudiólogos, psicopedagogos e educadores, em contextos clínicos, escolares e interdisciplinares.

Apesar dos achados promissores, o estudo apresenta limitações que devem ser mencionadas. A amostra foi reduzida e restrita a uma única escola privada no Nordeste brasileiro, o que limita a generalização dos resultados para outras regiões, realidades escolares e contextos socioeconômicos. Além disso, optou-se pela coleta em contexto escolar por conveniência, o que facilitou o acesso e a padronização da aplicação, mas não reflete o contexto clínico original para o qual o SPRouT foi desenvolvido. Embora o instrumento tenha sido concebido para uso em ambientes clínicos, especialmente por pediatras, este estudo utilizou uma amostra escolar, o que representa uma limitação em termos de adequação ao público-alvo original e aplicabilidade prática direta.

Dessa forma, recomenda-se que futuras pesquisas incluam amostras clínicas e contextos de atendimento em saúde, de modo a ampliar a evidência de validade do instrumento em diferentes ambientes de aplicação. Por fim, a amostra incluiu crianças com e sem suspeitas de dificuldades de aprendizagem, sem confirmação diagnóstica formal, introduzindo variação nosológica não controlada. Além disso, algumas crianças não completaram todos os instrumentos, por dificuldades atencionais ou baixa colaboração, reduzindo o número de respostas válidas em algumas análises.

## CONCLUSÃO

Apesar de seu caráter exploratório, o estudo forneceu evidências iniciais de validade convergente da versão brasileira do SPRouT, considerando suas relações com construtos relacionados ao desenvolvimento da leitura. Adicionalmente, os resultados indicam seu potencial preditivo, embora estudos longitudinais e com amostras clínicas sejam necessários para confirmação dessa propriedade. Recomenda-se, ainda, a investigação de outras fontes de evidências de validade e precisão (por exemplo, consistência interna e teste-reteste) por meio de amostras ampliadas e mais diversificadas.
